# [3-(5-Nitro-2-fur­yl)-1-phenyl-1*H*-pyrazol-4-yl](phen­yl)methanone

**DOI:** 10.1107/S1600536810015199

**Published:** 2010-04-30

**Authors:** Jia Hao Goh, Hoong-Kun Fun, Nithinchandra, B. Kalluraya, N. Satheesh Rai

**Affiliations:** a X-ray Crystallography Unit, School of Physics, Universiti Sains Malaysia, 11800 USM, Penang, Malaysia; b Department of Studies in Chemistry, Mangalore University, Mangalagangotri, Mangalore 574 199, India

## Abstract

In the title pyrazole compound, C_20_H_13_N_3_O_4_, an intra­molecular C—H⋯O hydrogen bond generates a seven-membered ring, producing an *S*(7) ring motif. The essentially planar furan and pyrazole rings [maximum deviations of 0.002 (1) and 0.007 (1) Å, respectively] are coplanar with each other, forming a dihedral angle of 3.06 (10)°. The pyrazole ring forms dihedral angles of 8.51 (9) and 56.81 (9)° with the two benzene rings. The nitro group is coplanar with the attached furan ring, as indicated by the dihedral angle of 2.5 (3)°. In the crystal packing, inter­molecular C—H⋯O hydrogen bonds link adjacent mol­ecules into two-mol­ecule-wide chains along the *a* axis. The crystal packing is further stabilized by weak inter­molecular C—H⋯π and π–π inter­actions [centroid–centroid distance = 3.4441 (10) Å].

## Related literature

For general background to and applications of the title compound, see: Kalluraya *et al.* (1994 ▶); Rai & Kalluraya (2006 ▶); Rai *et al.* (2008 ▶); Sridhar & Perumal (2003 ▶). For graph-set descriptions of hydrogen-bond ring motifs, see: Bernstein *et al.* (1995 ▶). For closely related structures, see: Goh *et al.* (2009**a* ▶,b* ▶). For the stability of the temperature controller used for the data collection, see: Cosier & Glazer (1986 ▶).
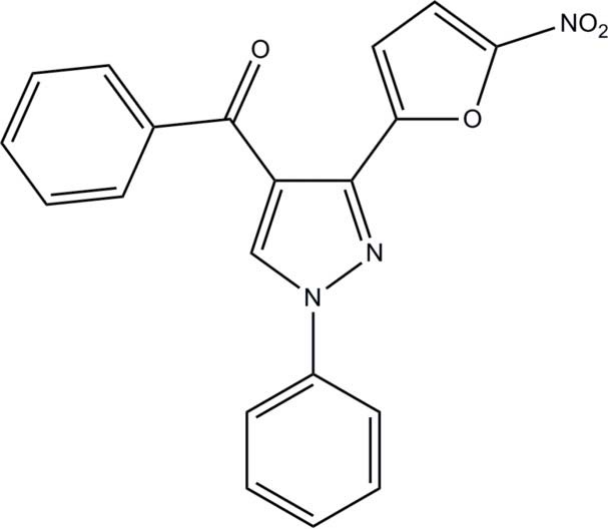


## Experimental

### 

#### Crystal data

C_20_H_13_N_3_O_4_
*M* *_r_* = 359.33
Monoclinic, 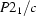

*a* = 11.2946 (12) Å
*b* = 6.9755 (8) Å
*c* = 22.7064 (18) Å
β = 115.015 (4)°
*V* = 1621.1 (3) Å^3^
*Z* = 4
Mo *K*α radiation
μ = 0.11 mm^−1^
*T* = 100 K
0.24 × 0.15 × 0.07 mm

#### Data collection

Bruker APEXII DUO CCD area-detector diffractometer
Absorption correction: multi-scan (*SADABS*; Bruker, 2009 ▶) *T*_min_ = 0.976, *T*_max_ = 0.992
17931 measured reflections
4725 independent reflections
2991 reflections with *I* > 2σ(*I*)
*R*_int_ = 0.056

#### Refinement

*R*[*F* ^2^ > 2σ(*F* ^2^)] = 0.051
*wR*(*F* ^2^) = 0.129
*S* = 1.02
4725 reflections
296 parameters
All H-atom parameters refined
Δρ_max_ = 0.37 e Å^−3^
Δρ_min_ = −0.24 e Å^−3^

### 

Data collection: *APEX2* (Bruker, 2009 ▶); cell refinement: *SAINT* (Bruker, 2009 ▶); data reduction: *SAINT*; program(s) used to solve structure: *SHELXTL* (Sheldrick, 2008 ▶); program(s) used to refine structure: *SHELXTL*; molecular graphics: *SHELXTL*; software used to prepare material for publication: *SHELXTL* and *PLATON* (Spek, 2009 ▶).

## Supplementary Material

Crystal structure: contains datablocks global, I. DOI: 10.1107/S1600536810015199/tk2653sup1.cif

Structure factors: contains datablocks I. DOI: 10.1107/S1600536810015199/tk2653Isup2.hkl

Additional supplementary materials:  crystallographic information; 3D view; checkCIF report

## Figures and Tables

**Table 1:** Hydrogen-bond geometry (Å, °) *Cg*1 is the centroid of the C15–C20 benzene ring.

*D*—H⋯*A*	*D*—H	H⋯*A*	*D*⋯*A*	*D*—H⋯*A*
C2—H2*A*⋯O4^i^	0.98 (2)	2.45 (2)	3.190 (3)	131.8 (17)
C11—H11*A*⋯O2	0.98 (3)	2.23 (3)	2.950 (3)	130 (2)
C14—H14*A*⋯O3^ii^	0.96 (2)	2.43 (2)	3.379 (3)	169.3 (17)
C18—H18*A*⋯*Cg*1^iii^	0.96 (2)	2.96 (2)	3.671 (2)	132.0 (16)

Symmetry codes: (i) 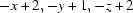; (ii) 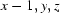; (iii) 
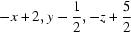
.

**Fig. 1.**
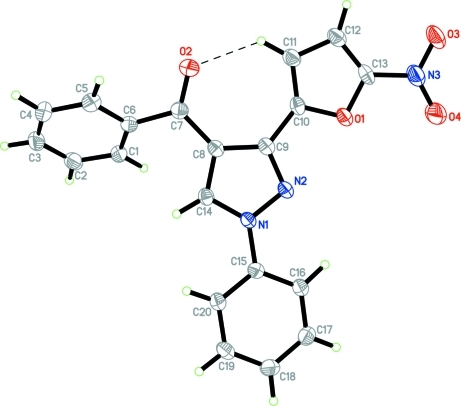
The molecular structure of the title compound, showing 50% probability displacement ellipsoids for non-H atoms and the atom-numbering scheme. An intramolecular hydrogen bond is shown as dashed line.

**Fig. 2.**
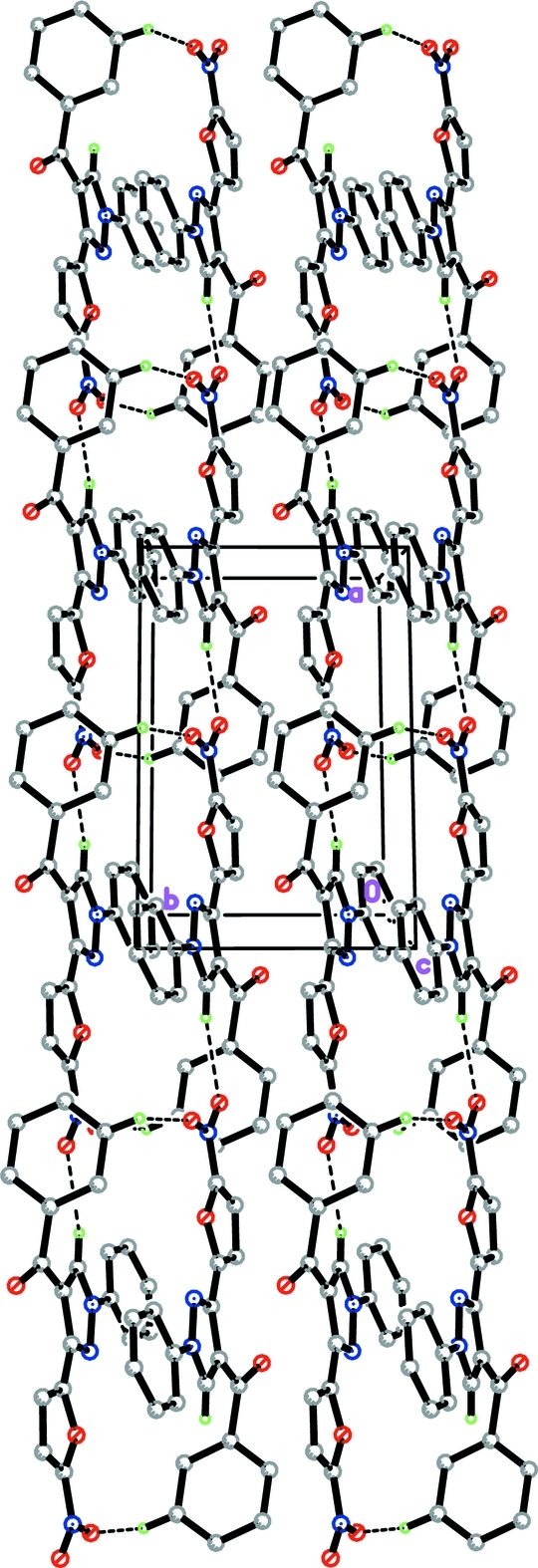
The crystal packing of the title compound, viewed along the c axis, showing two-molecule-wide chains along the a axis. Hydrogen atoms not involved in intermolecular interactions (dashed lines) have been omitted for clarity.

## supplementary crystallographic information

### Comment

Pyrazole derivatives are in general well-known nitrogen-containing heterocyclic compounds and various procedures have been developed for their synthesis (Rai & Kalluraya, 2006). The chemistry of pyrazole derivatives has been the subject of much interest due to their importance for various applications, and their widespread potential and proven biological and pharmacological activities (Rai *et al.*, 2008). Steroids containing pyrazole moiety are of interest as psychopharmacological agents. Some alkyl- and aryl- substituted pyrazoles have sharply pronounced sedative action on the central nervous system. Certain alkyl pyrazoles show significant bacteriostatic, bacteriocidal, fungicidal, analgesic and anti-pyretic activities (Sridhar & Perumal, 2003). In continuation of our studies on 1,3-dipolar cyclo-addition reactions of sydnones with dipolarophiles carrying a nitrofuran or a nitrothiophene moiety (Kalluraya *et al.*, 1994), we herein report the crystal structure of the title pyrazole compound.

In the title compound, Fig. 1, an intramolecular C11—H11A···O2 hydrogen bond (Table 1) generates a seven-membered ring, producing an *S*(7) ring motif (Bernstein *et al.*, 1995). The furan (C10-C13/O1) and pyrazole (C8/C9/N2/N1/C14) rings are essentially planar, with maximum deviations of 0.002 (1) and -0.007 (1) Å, respectively, at atoms C12 and N2. These two rings are coplanar to one another, as indicated by the dihedral angle formed between them of 3.06 (10)°. The pyrazole ring is inclined at dihedral angles of 56.81 (9) and 8.51 (9)°, respectively, with the C1-C6 and C15-C20 benzene rings. The nitro group is coplanar with the attached furan ring, making a dihedral angle of 2.5 (3)°. The bond lengths and angles agree well with those closely related pyrazole structures (Goh *et al.*, 2009*a,b*).

In the crystal packing, intermolecular C2—H2A···O4 and C14—H14A···O3 hydrogen bonds (Table 1) link adjacent molecules into two-molecule-wide chains along the *a* axis (Fig. 2). The crystal packing is further stabilized by weak intermolecular C18—H18A···*Cg*1 (Table 1) and *Cg*2···*Cg*2 interactions [*Cg*2···*Cg*2^i^ = 3.4441 (10) Å where *Cg*1 and *Cg*2 are the centroids of the C15-C20 benzene and pyrazole rings, respectively].

### Experimental

3-Phenylsydnone (0.01 mol) and 1-phenyl-3-(5-nitro-2-furyl)-2-propyn-1-one (0.01 mol) were dissolved in dry xylene (10 ml) and refluxed for 4 h. After completion of the reaction, the solvent was removed by distillation under reduced pressure. The crude product obtained was purified by recrystallization from a mixture of ethanol and DMF. The solid obtained was collected by filtration, washed with ethanol and dried. Single crystals suitable for X-ray analysis were obtained from a 1:2 mixture of ethanol and DMF by slow evaporation.

### Refinement

All the H atoms were located from difference Fourier map [range of C—H = 0.933 (18)–1.00 (3) Å] and allowed to refine freely.

### Crystal data

**Table:**

C_20_H_13_N_3_O_4_	*F*(000) = 744
*M**_r_* = 359.33	*D*_x_ = 1.472 Mg m^−^^3^
Monoclinic, *P*2_1_/*c*	Mo *K*α radiation, λ = 0.71073 Å
Hall symbol: -P 2ybc	Cell parameters from 2349 reflections
*a* = 11.2946 (12) Å	θ = 3.5–26.3°
*b* = 6.9755 (8) Å	µ = 0.11 mm^−^^1^
*c* = 22.7064 (18) Å	*T* = 100 K
β = 115.015 (4)°	Block, brown
*V* = 1621.1 (3) Å^3^	0.24 × 0.15 × 0.07 mm
*Z* = 4	

### Data collection

**Table:**

Bruker APEXII DUO CCD area-detector diffractometer	4725 independent reflections
Radiation source: fine-focus sealed tube	2991 reflections with *I* > 2σ(*I*)
graphite	*R*_int_ = 0.056
φ and ω scans	θ_max_ = 30.0°, θ_min_ = 2.0°
Absorption correction: multi-scan (*SADABS*; Bruker, 2009)	*h* = −15→15
*T*_min_ = 0.976, *T*_max_ = 0.992	*k* = −9→9
17931 measured reflections	*l* = −31→31

### Refinement

**Table:**

Refinement on *F*^2^	Primary atom site location: structure-invariant direct methods
Least-squares matrix: full	Secondary atom site location: difference Fourier map
*R*[*F*^2^ > 2σ(*F*^2^)] = 0.051	Hydrogen site location: inferred from neighbouring sites
*wR*(*F*^2^) = 0.129	All H-atom parameters refined
*S* = 1.02	*w* = 1/[σ^2^(*F*_o_^2^) + (0.0464*P*)^2^ + 0.6127*P*] where *P* = (*F*_o_^2^ + 2*F*_c_^2^)/3
4725 reflections	(Δ/σ)_max_ = 0.001
296 parameters	Δρ_max_ = 0.37 e Å^−^^3^
0 restraints	Δρ_min_ = −0.24 e Å^−^^3^

### Special details

**Table:**

Experimental. The crystal was placed in the cold stream of an Oxford Cryosystems Cobra open-flow nitrogen cryostat (Cosier & Glazer, 1986) operating at 100.0 (1)K.
Geometry. All esds (except the esd in the dihedral angle between two l.s. planes) are estimated using the full covariance matrix. The cell esds are taken into account individually in the estimation of esds in distances, angles and torsion angles; correlations between esds in cell parameters are only used when they are defined by crystal symmetry. An approximate (isotropic) treatment of cell esds is used for estimating esds involving l.s. planes.
Refinement. Refinement of F^2^ against ALL reflections. The weighted R-factor wR and goodness of fit S are based on F^2^, conventional R-factors R are based on F, with F set to zero for negative F^2^. The threshold expression of F^2^ > 2sigma(F^2^) is used only for calculating R-factors(gt) etc. and is not relevant to the choice of reflections for refinement. R-factors based on F^2^ are statistically about twice as large as those based on F, and R- factors based on ALL data will be even larger.

### Fractional atomic coordinates and isotropic or equivalent isotropic displacement parameters (Å^2^)

**Table:**

	*x*	*y*	*z*	*U*_iso_*/*U*_eq_	
O1	1.26743 (11)	0.74226 (19)	1.00753 (6)	0.0293 (3)	
O2	0.86733 (12)	0.95863 (19)	0.85528 (6)	0.0297 (3)	
O3	1.55123 (13)	0.7939 (2)	0.98584 (7)	0.0411 (4)	
O4	1.52027 (15)	0.6888 (3)	1.06817 (9)	0.0764 (7)	
N1	0.96246 (13)	0.6915 (2)	1.05307 (6)	0.0206 (3)	
N2	1.07625 (13)	0.6992 (2)	1.04603 (7)	0.0215 (3)	
N3	1.48065 (16)	0.7520 (3)	1.01308 (9)	0.0382 (4)	
C1	0.62475 (17)	0.6603 (3)	0.86532 (8)	0.0286 (4)	
C2	0.49042 (19)	0.6327 (4)	0.83427 (9)	0.0359 (5)	
C3	0.40950 (19)	0.7816 (4)	0.80076 (9)	0.0410 (5)	
C4	0.4611 (2)	0.9603 (4)	0.79806 (10)	0.0434 (6)	
C5	0.5954 (2)	0.9876 (3)	0.82757 (10)	0.0362 (5)	
C6	0.67801 (17)	0.8374 (3)	0.86116 (8)	0.0271 (4)	
C7	0.82209 (16)	0.8692 (3)	0.88763 (8)	0.0238 (4)	
C8	0.90414 (16)	0.7934 (2)	0.95280 (8)	0.0213 (3)	
C9	1.04207 (16)	0.7631 (2)	0.98559 (8)	0.0208 (3)	
C10	1.14371 (16)	0.7910 (2)	0.96325 (8)	0.0226 (4)	
C11	1.14713 (18)	0.8593 (3)	0.90780 (9)	0.0278 (4)	
C12	1.27856 (18)	0.8549 (3)	0.91693 (9)	0.0294 (4)	
C13	1.34517 (17)	0.7833 (3)	0.97709 (9)	0.0282 (4)	
C14	0.85851 (16)	0.7448 (2)	0.99826 (8)	0.0216 (3)	
C15	0.96295 (16)	0.6275 (2)	1.11294 (8)	0.0209 (3)	
C16	1.07647 (16)	0.5522 (3)	1.16079 (8)	0.0233 (4)	
C17	1.07388 (18)	0.4800 (3)	1.21724 (9)	0.0281 (4)	
C18	0.96039 (18)	0.4857 (3)	1.22655 (9)	0.0282 (4)	
C19	0.84901 (18)	0.5655 (3)	1.17896 (9)	0.0273 (4)	
C20	0.84850 (17)	0.6368 (3)	1.12181 (8)	0.0238 (4)	
H1A	0.6830 (18)	0.558 (3)	0.8899 (9)	0.024 (5)*	
H2A	0.458 (2)	0.505 (3)	0.8376 (11)	0.047 (7)*	
H3A	0.315 (2)	0.757 (3)	0.7776 (11)	0.047 (6)*	
H4A	0.406 (2)	1.071 (4)	0.7748 (12)	0.057 (7)*	
H5A	0.635 (2)	1.110 (4)	0.8263 (11)	0.048 (7)*	
H11A	1.071 (2)	0.900 (4)	0.8687 (12)	0.061 (7)*	
H12A	1.319 (2)	0.895 (3)	0.8890 (10)	0.041 (6)*	
H14A	0.7722 (19)	0.741 (3)	0.9963 (9)	0.028 (5)*	
H16A	1.1530 (18)	0.549 (3)	1.1519 (9)	0.025 (5)*	
H17A	1.150 (2)	0.425 (3)	1.2493 (10)	0.039 (6)*	
H18A	0.9566 (19)	0.433 (3)	1.2648 (10)	0.034 (5)*	
H19A	0.7709 (17)	0.575 (3)	1.1840 (8)	0.022 (5)*	
H20A	0.7707 (18)	0.686 (3)	1.0903 (9)	0.022 (5)*	

### Atomic displacement parameters (Å^2^)

**Table:**

	*U*^11^	*U*^22^	*U*^33^	*U*^12^	*U*^13^	*U*^23^
O1	0.0199 (6)	0.0361 (7)	0.0344 (7)	0.0003 (5)	0.0140 (5)	0.0054 (6)
O2	0.0304 (7)	0.0320 (7)	0.0293 (6)	−0.0019 (6)	0.0152 (5)	0.0027 (6)
O3	0.0260 (7)	0.0555 (10)	0.0492 (8)	−0.0051 (7)	0.0230 (6)	−0.0074 (7)
O4	0.0283 (8)	0.1271 (18)	0.0727 (12)	0.0171 (10)	0.0201 (8)	0.0604 (13)
N1	0.0185 (7)	0.0227 (7)	0.0226 (7)	−0.0021 (6)	0.0106 (5)	−0.0021 (6)
N2	0.0184 (6)	0.0232 (7)	0.0252 (7)	−0.0015 (6)	0.0115 (5)	−0.0030 (6)
N3	0.0244 (8)	0.0450 (11)	0.0475 (10)	0.0010 (7)	0.0176 (7)	0.0061 (9)
C1	0.0235 (9)	0.0393 (11)	0.0230 (8)	0.0008 (8)	0.0098 (7)	0.0008 (8)
C2	0.0274 (10)	0.0548 (14)	0.0264 (9)	−0.0065 (10)	0.0125 (8)	−0.0022 (10)
C3	0.0222 (9)	0.0738 (17)	0.0254 (9)	0.0009 (10)	0.0086 (8)	0.0023 (10)
C4	0.0268 (10)	0.0670 (16)	0.0350 (10)	0.0134 (11)	0.0116 (8)	0.0175 (11)
C5	0.0324 (10)	0.0443 (13)	0.0337 (10)	0.0069 (10)	0.0157 (8)	0.0113 (9)
C6	0.0240 (8)	0.0385 (11)	0.0205 (8)	0.0027 (8)	0.0110 (7)	0.0013 (8)
C7	0.0255 (8)	0.0242 (9)	0.0232 (8)	0.0008 (7)	0.0117 (7)	−0.0024 (7)
C8	0.0214 (8)	0.0199 (8)	0.0242 (8)	−0.0021 (7)	0.0112 (6)	−0.0029 (7)
C9	0.0216 (8)	0.0172 (8)	0.0246 (8)	−0.0027 (7)	0.0108 (6)	−0.0050 (7)
C10	0.0189 (8)	0.0216 (8)	0.0271 (8)	−0.0021 (7)	0.0095 (7)	−0.0040 (7)
C11	0.0258 (9)	0.0337 (10)	0.0276 (9)	−0.0023 (8)	0.0148 (7)	−0.0026 (8)
C12	0.0276 (9)	0.0352 (11)	0.0303 (9)	−0.0065 (8)	0.0171 (7)	−0.0070 (8)
C13	0.0193 (8)	0.0301 (10)	0.0387 (10)	−0.0020 (7)	0.0157 (7)	−0.0026 (8)
C14	0.0197 (8)	0.0214 (8)	0.0239 (8)	0.0003 (7)	0.0095 (6)	−0.0030 (7)
C15	0.0225 (8)	0.0196 (8)	0.0221 (7)	−0.0030 (7)	0.0108 (6)	−0.0027 (7)
C16	0.0207 (8)	0.0245 (9)	0.0257 (8)	−0.0017 (7)	0.0106 (7)	−0.0032 (7)
C17	0.0270 (9)	0.0287 (10)	0.0251 (8)	−0.0012 (8)	0.0077 (7)	0.0007 (8)
C18	0.0327 (10)	0.0291 (10)	0.0240 (8)	−0.0073 (8)	0.0131 (7)	−0.0024 (8)
C19	0.0254 (9)	0.0305 (10)	0.0315 (9)	−0.0041 (8)	0.0173 (7)	−0.0042 (8)
C20	0.0204 (8)	0.0248 (9)	0.0262 (8)	−0.0013 (7)	0.0099 (7)	−0.0020 (7)

### Geometric parameters (Å, °)

**Table:**

O1—C13	1.358 (2)	C7—C8	1.472 (2)
O1—C10	1.375 (2)	C8—C14	1.377 (2)
O2—C7	1.228 (2)	C8—C9	1.430 (2)
O3—N3	1.233 (2)	C9—C10	1.451 (2)
O4—N3	1.219 (2)	C10—C11	1.362 (2)
N1—C14	1.353 (2)	C11—C12	1.410 (3)
N1—N2	1.3616 (18)	C11—H11A	0.98 (2)
N1—C15	1.429 (2)	C12—C13	1.346 (3)
N2—C9	1.335 (2)	C12—H12A	0.96 (2)
N3—C13	1.414 (2)	C14—H14A	0.96 (2)
C1—C2	1.390 (3)	C15—C16	1.387 (2)
C1—C6	1.395 (3)	C15—C20	1.391 (2)
C1—H1A	0.97 (2)	C16—C17	1.389 (2)
C2—C3	1.380 (3)	C16—H16A	0.968 (18)
C2—H2A	0.98 (2)	C17—C18	1.385 (3)
C3—C4	1.388 (3)	C17—H17A	0.94 (2)
C3—H3A	0.99 (2)	C18—C19	1.382 (3)
C4—C5	1.389 (3)	C18—H18A	0.96 (2)
C4—H4A	1.00 (3)	C19—C20	1.387 (2)
C5—C6	1.396 (3)	C19—H19A	0.939 (18)
C5—H5A	0.97 (2)	C20—H20A	0.933 (18)
C6—C7	1.494 (2)		
			
C13—O1—C10	104.57 (13)	C8—C9—C10	130.30 (15)
C14—N1—N2	112.10 (13)	C11—C10—O1	110.18 (14)
C14—N1—C15	127.88 (14)	C11—C10—C9	134.91 (16)
N2—N1—C15	120.00 (13)	O1—C10—C9	114.89 (14)
C9—N2—N1	104.88 (13)	C10—C11—C12	107.23 (16)
O4—N3—O3	124.47 (17)	C10—C11—H11A	125.5 (14)
O4—N3—C13	119.14 (17)	C12—C11—H11A	127.2 (14)
O3—N3—C13	116.38 (17)	C13—C12—C11	105.03 (16)
C2—C1—C6	119.85 (19)	C13—C12—H12A	124.0 (13)
C2—C1—H1A	121.2 (11)	C11—C12—H12A	131.0 (13)
C6—C1—H1A	118.9 (11)	C12—C13—O1	112.99 (15)
C3—C2—C1	120.1 (2)	C12—C13—N3	130.37 (17)
C3—C2—H2A	123.0 (14)	O1—C13—N3	116.60 (16)
C1—C2—H2A	116.9 (13)	N1—C14—C8	107.64 (15)
C2—C3—C4	120.51 (19)	N1—C14—H14A	120.4 (11)
C2—C3—H3A	118.6 (14)	C8—C14—H14A	132.0 (11)
C4—C3—H3A	120.8 (14)	C16—C15—C20	120.80 (16)
C3—C4—C5	119.7 (2)	C16—C15—N1	119.63 (14)
C3—C4—H4A	122.8 (14)	C20—C15—N1	119.55 (15)
C5—C4—H4A	117.5 (14)	C15—C16—C17	119.16 (16)
C4—C5—C6	120.2 (2)	C15—C16—H16A	117.3 (11)
C4—C5—H5A	122.1 (13)	C17—C16—H16A	123.6 (11)
C6—C5—H5A	117.7 (14)	C18—C17—C16	120.84 (17)
C1—C6—C5	119.62 (17)	C18—C17—H17A	119.5 (13)
C1—C6—C7	122.14 (17)	C16—C17—H17A	119.6 (13)
C5—C6—C7	118.10 (18)	C19—C18—C17	119.16 (17)
O2—C7—C8	122.64 (16)	C19—C18—H18A	119.1 (12)
O2—C7—C6	119.53 (15)	C17—C18—H18A	121.7 (12)
C8—C7—C6	117.83 (15)	C18—C19—C20	121.20 (17)
C14—C8—C9	104.11 (14)	C18—C19—H19A	121.7 (11)
C14—C8—C7	124.45 (15)	C20—C19—H19A	117.1 (11)
C9—C8—C7	131.35 (15)	C19—C20—C15	118.82 (16)
N2—C9—C8	111.25 (14)	C19—C20—H20A	118.7 (11)
N2—C9—C10	118.45 (15)	C15—C20—H20A	122.5 (11)
			
C14—N1—N2—C9	1.32 (18)	C8—C9—C10—O1	−178.34 (16)
C15—N1—N2—C9	179.83 (15)	O1—C10—C11—C12	−0.2 (2)
C6—C1—C2—C3	−1.6 (3)	C9—C10—C11—C12	178.25 (19)
C1—C2—C3—C4	−0.4 (3)	C10—C11—C12—C13	0.3 (2)
C2—C3—C4—C5	2.1 (3)	C11—C12—C13—O1	−0.3 (2)
C3—C4—C5—C6	−1.6 (3)	C11—C12—C13—N3	−177.8 (2)
C2—C1—C6—C5	2.1 (3)	C10—O1—C13—C12	0.2 (2)
C2—C1—C6—C7	−173.56 (16)	C10—O1—C13—N3	178.07 (16)
C4—C5—C6—C1	−0.5 (3)	O4—N3—C13—C12	178.3 (2)
C4—C5—C6—C7	175.34 (18)	O3—N3—C13—C12	−0.8 (3)
C1—C6—C7—O2	135.87 (18)	O4—N3—C13—O1	0.9 (3)
C5—C6—C7—O2	−39.8 (2)	O3—N3—C13—O1	−178.27 (17)
C1—C6—C7—C8	−45.1 (2)	N2—N1—C14—C8	−1.0 (2)
C5—C6—C7—C8	139.15 (17)	C15—N1—C14—C8	−179.35 (16)
O2—C7—C8—C14	159.65 (17)	C9—C8—C14—N1	0.24 (19)
C6—C7—C8—C14	−19.3 (3)	C7—C8—C14—N1	−176.58 (16)
O2—C7—C8—C9	−16.2 (3)	C14—N1—C15—C16	170.60 (17)
C6—C7—C8—C9	164.80 (17)	N2—N1—C15—C16	−7.6 (2)
N1—N2—C9—C8	−1.14 (18)	C14—N1—C15—C20	−7.7 (3)
N1—N2—C9—C10	178.84 (14)	N2—N1—C15—C20	174.08 (15)
C14—C8—C9—N2	0.58 (19)	C20—C15—C16—C17	2.0 (3)
C7—C8—C9—N2	177.09 (17)	N1—C15—C16—C17	−176.24 (16)
C14—C8—C9—C10	−179.40 (17)	C15—C16—C17—C18	−1.1 (3)
C7—C8—C9—C10	−2.9 (3)	C16—C17—C18—C19	−0.4 (3)
C13—O1—C10—C11	0.0 (2)	C17—C18—C19—C20	1.1 (3)
C13—O1—C10—C9	−178.77 (15)	C18—C19—C20—C15	−0.2 (3)
N2—C9—C10—C11	−176.7 (2)	C16—C15—C20—C19	−1.3 (3)
C8—C9—C10—C11	3.2 (3)	N1—C15—C20—C19	176.91 (16)
N2—C9—C10—O1	1.7 (2)		

### Hydrogen-bond geometry (Å, °)

**Table:**

Cg1 is the centroid of the C15–C20 benzene ring.

**Table:**

*D*—H···*A*	*D*—H	H···*A*	*D*···*A*	*D*—H···*A*
C2—H2A···O4^i^	0.98 (2)	2.45 (2)	3.190 (3)	131.8 (17)
C11—H11A···O2	0.98 (3)	2.23 (3)	2.950 (3)	130 (2)
C14—H14A···O3^ii^	0.96 (2)	2.43 (2)	3.379 (3)	169.3 (17)
C18—H18A···Cg1^iii^	0.96 (2)	2.96 (2)	3.671 (2)	132.0 (16)

Symmetry codes: (i) −*x*+2, −*y*+1, −*z*+2; (ii) *x*−1, *y*, *z*; (iii) −*x*+2, *y*−1/2, −*z*+5/2.
